# α-Synuclein and neuronal cell death

**DOI:** 10.1186/1750-1326-4-9

**Published:** 2009-02-04

**Authors:** Mark R Cookson

**Affiliations:** 1 Laboratory of Neurogenetics, National Institute on Aging, NIH. Building 35, Room 1A116, MSC 3707, 35 Convent Drive, Bethesda, MD 20982-3707, USA

## Abstract

α-Synuclein is a small protein that has special relevance for understanding Parkinson disease and related disorders. Not only is α-synuclein found in Lewy bodies characteristic of Parkinson disease, but also mutations in the gene for α-synuclein can cause an inherited form of Parkinson disease and expression of normal α-synuclein can increase the risk of developing Parkinson disease in sporadic, or non-familial, cases. Both sporadic and familial Parkinson disease are characterized by substantial loss of several groups of neurons, including the dopaminergic cells of the substantia nigra that are the target of most current symptomatic therapies. Therefore, it is predicted that α-synuclein, especially in its mutant forms or under conditions where its expression levels are increased, is a toxic protein in the sense that it is associated with an increased rate of neuronal cell death. This review will discuss the experimental contexts in which α-synuclein has been demonstrated to be toxic. I will also outline what is known about the mechanisms by which α-synuclein triggers neuronal damage, and identify some of the current gaps in our knowledge about this subject. Finally, the therapeutic implications of toxicity of α-synuclein will be discussed.

## 

All neurodegenerative diseases share the common phenomenon that neurons, usually relatively specific groups, are lost progressively as the disease develops. In some cases, we can provide partial relief for patients by treating some of their symptoms. However, because we don't understand the underling mechanisms of why neurons die, degeneration continues inexorably and old symptoms often become unresponsive while new ones arrive. At the end of the disease process, we are left with only a few clues about what might have happened based on what we can glean from the pathology of the disease using *post mortem *samples. In general, the root cause of neurodegeneration remains obscure although rare genetic variants are helpful in that we can be certain that an inherited mutation acts as the trigger for disease in that specific family.

Here, I will discuss cell loss related to Parkinson disease (PD) in the context of one protein, α-synuclein, that has several links to the disorder. In doing so, I will outline what we know about the ways in which a protein can lead to cell death. Before doing so, it is worth discussing what PD is, and what it isn't.

## Cell death in PD

It is very commonly said that PD is the second most common neurodegenerative disease and results from a loss of dopamine neurons. The first fact is dull and the second tells only part of the story. It is true that PD patients have a substantial loss of dopamine in the striatum resulting from a relatively selective loss of dopaminergic projection neurons in the substantia nigra pars compacta. Both biochemical measures and imaging modalities suggest that at least a 70% decrease in striatal dopamine occurs before the onset of clinical parkinsonism and progresses over time [1]. These estimates of the extent of striatal dopamine depletion, combined with the observation that the majority of dopaminergic neurons are lost by the end of the disease process, imply that there is substantial cell death throughout the PD disease process. It is not possible to show this directly, but measurements of nigral cell numbers in neurologically normal people and in non-human primates reveal a slow progressive loss of dopamine neurons with age [2]. In this view, parkinsonism is an accelerated, but still slow, cell death phenotype that would normally be seen with aging [3].

However, while there is relative vulnerability of dopaminergic neurons in the substantia nigra [4], not all dopamine cells are affected in PD. For example, although dopaminergic neurons in the ventral tegmental area that project to the nucleus accumbens do degenerate [5], compared to the dopaminergic neurons in the substantia nigra pars compacta these cells are relatively spared [6,7].

Furthermore, not all affected neurons in PD are dopaminergic. Non-motor symptoms are a serious problem for many PD patients and are often untreated by replacement therapy with L-DOPA (3,4-dihydroxy-L-phenylalanine) [8]. A good example of non-dopaminergic cells that degenerate in PD is the cholinergic neurons in the dorsal vagal nucleus [9]. It has been suggested that involvement of non-nigral regions underlies the complex clinical picture in PD [10]. Therefore, although there is some specificity to cell death in PD, there is no absolute selectivity for any specific neurotransmitter group or anatomic region. It is also important to note that loss of nigral neurons occurs in diverse pathological situations [4] and that on its own, nigral cell loss defines the clinical term parkinsonism, not Parkinson disease.

This distinction is also important when discussing the other major pathological event in PD that appears alongside cell death, the formation of Lewy bodies and Lewy neurites. Lewy bodies are intracellular deposits of proteins and lipids [11] that were traditionally stained with eosin but now are more sensitively recognized by antibodies to specific marker proteins [12]. Using electron microscopy, Lewy bodies are fibrillar structures with a recognizable core and halo [13]. The range of Lewy pathology is now recognized as encompassing many regions of the diseased brain [14] including, for example, the olfactory bulb, raphe nucleus, locus coeruleus and the basal nucleus of Meynert. Additionally, some reports suggest that the nigra is not the first place where Lewy bodies form [15]. How this relates to the extent of cell loss in each region is not well defined. Lewy bodies are also seen in dementia with Lewy bodies (DLB, also known as Diffuse Lewy body Disease or DLBD), suggesting that PD and DLBD are related to one another by shared pathology and maybe by shared etiology.

Therefore, PD is a disease where substantial cell loss in the nigra occurs alongside the formation of Lewy bodies. Neither cell loss nor Lewy bodies is absolutely specific for PD but both are required for a diagnosis of PD under current definitions [16]. This review will focus on cell death, but it is important to understand a little more about the most commonly used marker for Lewy bodies; α-synuclein.

## α-Synuclein is a marker of the PD process

The first member of the family of proteins for which α-synuclein is named was cloned from the neuromuscular junction of the electric eel [17]. Antibodies against that protein labeled both synapses and nuclei, leading to the naming of synuclein. A related protein was cloned from zebra finch as a protein upregulated during the process of song learning, a period of enormous synaptic plasticity [18]. In humans, there are three synuclein family members (α-,β-,γ-) and all synuclein genes are relatively well conserved both within and between species [19]. The synuclein genes are specific to the vertebrate lineage in that neither single cell organisms (including yeast) nor invertebrates (*Drosophila melanogaster*, *Caenorhabditis elegans*) have any apparent synuclein homologue. Additionally, primate α-synuclein sequences differ from other vertebrate synucleins by a substitution of Alanine for a Threonine at position 53 [20]. These two interesting facts about the evolutionary relationships in the synuclein family are important for understanding some of the experimental systems where synuclein has been explored.

The normal function of α-synuclein is poorly understood. Although it is expressed at high levels in the brain, relatively specifically within neurons, it is also found in other tissues, e.g., hematopoietic cells [21,22]. α-Synuclein can bind to lipids [23] and, in neurons, is associated with presynaptic vesicles [24,25] and the plasma membrane, possibly via lipid rafts [26]. The association of α-synuclein with vesicles is modulated by synaptic activity where the protein dissociates from vesicles after electrical stimulation of the neuron and only slowly re-associates [27]. However, α-synuclein knockout mice show only subtle abnormalities in neurotransmission [28-30], suggesting that α-synuclein plays a non-essential function at the synapse. There is some evidence that such a modulatory role may be more important under conditions where reactive oxygen species or nitric oxide are present [31,32], although the mechanism(s) are not yet fully defined.

In the normal brain, α-synuclein immunostaining reveals a diffuse pattern of reactivity throughout the neuropil that would be consistent with a predominantly synaptic localization [25]. However, in PD brains, α-synuclein antibodies strongly stain Lewy bodies [33] and Lewy neurites [34]. Because of this sensitivity, α-synuclein staining is now more commonly used than eosin or ubiquitin staining for these structures. Biochemical analyses have shown that α-synuclein is a major protein component of Lewy bodies and may be part of the fibrillar structure of these structures [35]. The deposited, pathological forms of α-synuclein are aggregated and show lower solubility than the normal protein [36] and may be modified post-translationally by truncation, nitration, ubiquitylation and phosphorylation [37-40].

Therefore, α-synuclein protein deposition into Lewy bodies is a marker of the PD disease state. However, because we require Lewy bodies for a PD diagnosis this isn't an especially strong argument for involvement of α-synuclein in the disease process. It is also important to note that, although we cannot determine if Lewy bodies previously formed in the cells that eventually died, the individual neurons where Lewy bodies are found are the ones that have survived the disease process (though they still may be dysfunctional). Very recently, it has been shown that Lewy bodies form in functional dopaminergic neurons grafted in to brains of people with PD as a therapeutic intervention [41,42], although this is not always seen [43]. These were embryonic cells that remained apparently healthy and were functional after grafting, which suggests that there is the environment of the PD brain predisposes even young cells to form Lewy bodies.

In summary, the available evidence identifies α-synuclein as a marker of the PD/DLB process but do not prove that it has a causal role. The evidence that it does comes from a variety of human genetic studies.

## α-Synuclein can cause PD

A key discovery in understanding PD was the report that an A53T mutation in the α-synuclein gene was causal for dominantly inherited disease [44]. This was the first clear report that a Mendelian gene could be a cause of PD, which to that point had been thought of as a non-genetic disease. It is interesting that the first mutation found was A53T, i.e. a reversion of the human Alanine to the ancestral Threonine found in rodents and many other species. Since then, two other point mutations, A30P [45] and E46K [46], have been reported in different families. It is also important that while many cases are reported to have a phenotype of 'PD', in fact several patients in the A53T and E46K [46] families have a more diffuse involvement of synuclein deposition [47,48] and clinical features that presumably result from this degree of involvement of non-dopaminergic systems [49].

A second group of important cases have multiplications of the normal wild type allele of *SNCA*, the gene that encodes for the α-synuclein protein. Cases with *SNCA *duplication have a brainstem-predominant PD phenotype [50], while cases with a triplication have a Lewy body disease that again involves several brain regions [51,52]. Measurements of protein levels in triplication show the predicted doubling of α-synuclein in blood as well as increased levels and deposition of the protein in the cerebral cortex where Lewy bodies are found [21]. Therefore, even without sequence variants, α-synuclein dosage can be causal for Lewy body disease.

A third piece of genetic evidence comes from the reports common variants around the α-synuclein gene are associated with lifetime risk of sporadic PD. Both the promoter region, specifically the Rep1 polymorphic repeat [53], and polymorphisms towards the 3' end of the gene are associated with PD [54]. Although it is not known specifically how these risk variants influence lifetime incidence of PD, it seems likely that they increase α-synuclein protein levels in the brain.

Collectively, the human genetic data strongly support a causal role for α-synuclein in PD/DLBD. Whether Lewy bodies are causal or consequential is less clear, but they do support the idea that α-synuclein represents an important link between sporadic and inherited PD. The various lines of evidence identify α-synuclein as a potentially toxic protein, fulfilling the requirements of a causative agent in PD [55]. The question now is how, and in what context, is α-synuclein toxic, and can we do anything about it?

## Where and when is α-synuclein toxic?

Given that cell loss is a major event in human PD, combined with the evidence that α-synuclein plays a causal role in disease, it is reasonable to infer that α-synuclein is toxic to human neurons. The time course is likely to be protracted, with the most likely explanation that there is asynchronous cell death that results in a slow depletion of the populations of relatively vulnerable neurons. However, it is not possible to watch cells die in the human brain and so we have to turn to experimental models to confirm or refute the idea that α-synuclein is toxic.

Yeast models are probably the simplest system used to show that expression of human α-synuclein evokes toxic events. In growing and stationary phase cultures, increased expression of α-synuclein limits cell growth [56-65]. These experiments are extraordinarily useful in defining pathways that underpin the toxic effects of the protein. α-Synuclein toxicity has also been demonstrated in *Drosophila*, where dopaminergic neuron cell loss has been reported [66-73], although this result is a little controversial [74] and the effects are modest. The worm *C. elegans *can also be used to show that α-synuclein can damage dopamine neurons in an intact, *in vivo*, setting [75-80]. What links these three model systems is that they all show a detrimental effect of expression of α-synuclein in organisms where the protein is not normally present. One reading of this data is that, at least in terms of toxicity occurring over days to weeks, the normal function of the protein is probably not relevant.

A situation where α-synuclein is normally present is in mammalian cell culture models. Two commonly used systems are primary neurons, including dopaminergic cultures of the ventral midbrain, or neuroblastoma derived cell lines. Experiments showing the most substantial effects of α-synuclein include those where the protein is transiently expressed, e.g. from viral vectors [81-86], or expression is controlled from an inducible promoter system [87-89], although some authors have reported a lack of toxicity in similar circumstances [90]. In midbrain cultures, toxicity is higher for dopamine neurons than other cells [81], which may be relevant to the relative vulnerability of nigral neurons in PD. Some experiments show nicely that the difference between wild type and mutant protein is really a matter of dose and that at increasing expression levels, the normal protein becomes as toxic as the dominant mutants [89].

Although potentially useful in for understanding mechanisms, these cell-based models are taken out of their *in vivo *context and tend to show cell loss over a few days, compared to the predicted years of progress in the disease. A more intact approach is to express α-synuclein using transgenic technology in various parts of the mouse CNS. Some of these models show toxicity, particularly in the spinal cord, but nigral cell loss is absent or modest [91-97]. Several models do show accumulation and insolubility of α-synuclein [e.g., [36,91,93,98]], although whether true Lewy bodies are formed is uncertain. Therefore, most mouse models reported to date are better for understanding α-synuclein deposition than frank cellular toxicity. Why this is the case is unclear, but it is interesting that crossing transgenic models with mouse α-synuclein knockouts exacerbates phenotypes [99-101], suggesting that the presence of the murine protein limits damage in some undefined way. The lack of an ideal PD mouse model that more completely captures the human phenotype limits our current studies of α-synuclein toxicity. Though a goal worth pursuing, creation of such an ideal mouse model may be very challenging given the limitations of mouse lifespan and differences in physiology between mice and humans.

An alternate approach to traditional transgenics is to use viral vectors to deliver α-synuclein directly to the substantia nigra in mice [102], rats [103-106] or non-human primates [107-109]. In these approaches, a significant cell loss is noted along with deposition of α-synuclein protein. The extent of cell loss is less dramatic than in human PD and behavioral effects are similarly modest. However, the critical observation here is that α-synuclein can induce toxicity *in vivo *using vertebrate organisms, with a time course of several weeks, allowing for some dissection of mechanism.

Taken together, all of this evidence suggests that α-synuclein can induce toxicity in a variety of contexts, from simple organisms to dopamine neurons in the primate substantia nigra. It is less clear whether all of these situations are directly relevant to the human disease, where cell loss is probably more protracted, but as a practical matter such models at least afford an opportunity to examine mechanism(s) by which α-synuclein triggers neuronal death.

## Why is α-synuclein toxic?

**Figure 1:**
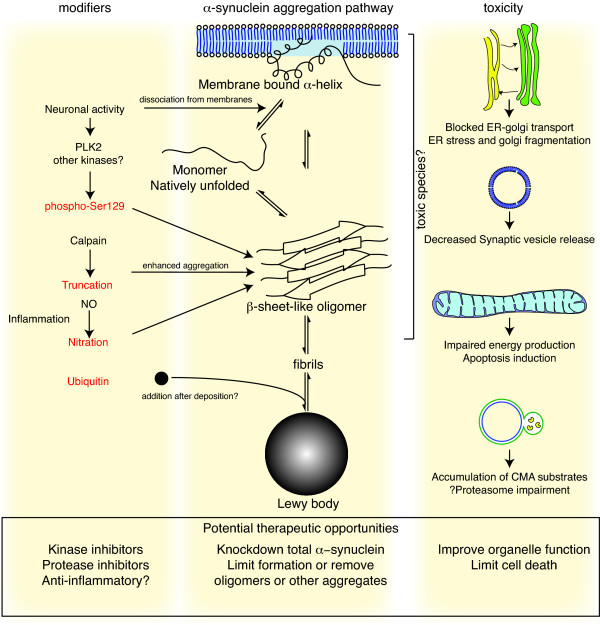
**Events in α-synuclein toxicity**. The central panel shows the major pathway for protein aggregation. Monomeric α-synuclein is natively unfolded in solution but can also bind to membranes in an α-helical form. It seems likely that these two species exist in equilibrium within the cell, although this is unproven. From *in vitro *work, it is clear that unfolded monomer can aggregate first into small oligomeric species that can be stabilized by β-sheet-like interactions and then into higher molecular weight insoluble fibrils. In a cellular context, there is some evidence that the presence of lipids can promote oligomer formation: α-synuclein can also form annular, pore-like structures that interact with membranes. The deposition of α-synuclein into pathological structures such as Lewy bodies is probably a late event that occurs in some neurons. On the left hand side are some of the known modifiers of this process. Electrical activity in neurons changes the association of α-synuclein with vesicles and may also stimulate polo-like kinase 2 (PLK2), which has been shown to phosphorylate α-synuclein at Ser129. Other kinases have also been proposed to be involved. As well as phosphorylation, truncation through proteases such as calpains, and nitration, probably through nitric oxide (NO) or other reactive nitrogen species that are present during inflammation, all modify synuclein such that it has a higher tendency to aggregate. The addition of ubiquitin (shown as a black spot) to Lewy bodies is probably a secondary process to deposition. On the right are some of the proposed cellular targets for α-synuclein mediated toxicity, which include (from top to bottom) ER-golgi transport, synaptic vesicles, mitochondria and lysosomes and other proteolytic machinery. In each of these cases, it is proposed that α-synuclein has detrimental effects, listed below each arrow, although at this time it is not clear if any of these are either necessary or sufficient for toxicity in neurons.

Some of the above model systems have been used to probe the mechanism(s) by which α-synuclein causes cell death. These can generally be sorted into aspects of the protein itself effects of the protein to the biological system (see figure 1). Appendix 1 highlights some of the key observations related to this critical question.

### Aspects of protein chemistry of α-synuclein and toxicity

α-Synuclein has a strong tendency to self-associate *in vitro *[110,111], and so a prime candidate for the underlying driving force for toxicity is the formation of aggregated species. One of the important questions about this idea is which species are present in cells/tissues. Oligomeric species can be isolated from cells [112-114] and from human [21] and mouse (both wild type and α-synuclein transgenic) brain [115]. In both cells and brain, oligomers are particularly found in membrane-enriched fractions [112,115], suggesting a possible influence of the lipid environment on oligomer formation. Higher molecular weight forms have also been found in some models [116], especially after oxidative stress [117] or exposure to inflammatory triggers in mice [100]. Deposited α-synuclein immunoreactivity has been seen in transgenic [91-97] or viral models [102-109]. However, the observation of aggregated α-synuclein by and of itself does not prove that aggregation is important; as discussed for Lewy bodies, all this proves is that deposition occurs, not that it is causal.

Some recent studies have attempted to answer this question, mainly using cell-based approaches. For example, some oligomeric forms of α-synuclein trigger calcium entry and toxicity in SY5Y cells [118]. Interestingly, different species show differential toxicity, suggesting that not all oligomers are created equal. However, the nature of this experiment is to add α-synuclein to the outside of the cell, which may or may not be relevant to the pathophysiological situation. As α-synuclein is intracellular, it seems more likely that the protein would form aggregate inside cells. The presence of fibrils in Lewy bodies would support this contention. However, α-synuclein can end up in the extracellular media [119] and it is possible that the conditions for aggregation might be more suitable in a milieu free of cells. The relevance of extracellular α-synuclein is an important question, raised also by the observation of Lewy bodies in grafted neurons [41,42] and the attendant hypothesis of 'host to graft transmission'.

Some studies have attempted to address whether intracellular aggregates of α-synuclein contribute to toxicity. For example, several imaging techniques shown that, in the context of a living cell, α-synuclein can form small oligomers, likely in an antiparallel configuration [114,120] and such oligomers can be associated with cell toxicity.

These approaches have been used to show that overexpression of heat shock proteins (Hsps) can mitigate both oligomer formation and toxicity [114,120,121]. *In vivo*, Hsps can prevent toxic effects of α-synuclein in yeast [59] and in flies [67]. Whether these studies constitute formal proof that aggregation is required for toxicity is unclear as there are other theoretical interpretations of the data. For example, a formal possibility is that monomeric α-synuclein is toxic and, thus, any protein binding the protein directly could limit toxicity. It should be stated that the mechanism(s) by which monomers of α-synuclein could be toxic are relatively unexplored but, equally, there is an absence of proof that aggregation is absolutely required for toxicity. Alternatively, Hsps could be limiting a detrimental event downstream of the initial aggregation and thus may neither represent evidence for or against the role of aggregation in α-synuclein toxicity. Interestingly, Hsp expression in the fly model decreases neuronal toxicity without any change in the number of α-synuclein positive inclusions [67].

Overall, these considerations show that α-synuclein is capable of protein aggregation and can be deposited into inclusion bodies of various forms *in vivo*, but that there is insufficient evidence that aggregation or deposition is either necessary or sufficient for toxicity. In fact, several lines of evidence show that toxicity can be dissociated from deposition, including; the observation in cells of toxicity without deposition in some models [81]; differential effects on toxicity and inclusions of various manipulations of α-synuclein in fly models [66,67]; and deposition of α-synuclein without clear toxic effects in some mouse models [e.g., [36]]. A key challenge for the field, therefore, is to understand whether protein aggregation is at all relevant for the toxic effects of α-synuclein. One way to potentially address this is to isolate various aggregated species of the protein and to express them within a neuron. This might be extraordinarily difficult from a technical standpoint and there is always possibility that the small aggregates would seed larger ones may confound interpretation. Another potential approach would be to develop reagents that limit the biological availability of specific aggregated species and use these to probe which agents are toxic in intact cells. As an example, recombinant single chain Fv antibody fragments against aggregated α-synuclein have been described [122,123] that might be helpful.

α-Synuclein has many additional properties as well as the tendency to aggregate. Some of the post-translational modifications that have been reported have also been explored as possible mediators of toxicity. For example, antibodies against α-synuclein phosphorylated at Ser129 are very good at identifying Lewy pathology in the human brain [38], suggesting either that Ser129 phosphorylation is a causal event for deposition or represents a common modification of the protein after it is deposited. Several groups have therefore made versions of α-synuclein that cannot be modified at this residue (S129A) or pseudo-phosphorylation mimics (S129D, S129E) and determined the toxic effects of expression. In *Drosophila *models, S129A is less toxic but has an increased tendency to form inclusion bodies compared to wild type protein [66]. The S129D phosphomimic has the opposite effect, i.e. increased toxicity but fewer inclusions. In contrast, similar experiments using viral overexpression in rats show the opposite result, namely that S129A greatly increases the toxic effects of expression [124]. In mammalian cell culture, S129A has a diminished tendency to form inclusion bodies [125].

At first glance, these results seem to suggest that the behavior of α-synuclein as it relates to toxicity is opposite in mammals compared to invertebrates where, it is important to note, the protein is not normally present. However, interpretation is complicated by several considerations. First, the expression levels of α-synuclein are critical for toxicity, which is shown by the human case where a difference in protein levels is 2-fold in the triplication cases and 1.5-fold in the duplication cases. Second, recent data suggests that the phosphomimic S129D/E α-synuclein variants have different biophysical properties compared to authentically phosphorylated wild type protein [126]. Overall, these considerations raise some important *caveats *about comparison of properties of α-synuclein in terms of concentration-dependent behaviors of the protein such as aggregation and toxicity.

One alternate approach to understand α-synuclein phosphorylation is to identify the kinase that mediates the phosphotransfer event. Casein kinase II and GRK2/5 have been shown to phosphorylate α-synuclein *in vitro *or in cells and work in yeast [64] and flies [66] respectively shows that they are at least active *in vivo*. More recently, the polo-like kinase family, specifically PLK2, have been shown to be active both *in vitro *and *in vivo *in generating pS129 α-synuclein [127]. What is interesting about PLK2 is that it is known to respond to neuronal activity [128], suggesting a possible link between neuronal phenotype and α-synuclein toxicity. However, it is not yet known in PLK2 inhibitors or gene knockout will limit the toxic effects of α-synuclein *in vivo*. Such experiments are feasible in several species as PLK2 homologues are present in mice and flies, and there is at least one polo kinase in yeast.

There are a number of other modifications of α-synuclein that have been reported and some of these are found more often in pathological circumstances than under normal conditions, such as nitration or truncation. Truncation of α-synuclein is associated with a higher tendency for aggregation [129-131]. Transgenic mice expressing truncated α-synuclein have substantial cell loss [101] although in at least one line, this is a developmental and not degenerative phenotype [132]. Again, because the window for toxicity is quite narrow, comparison between different lines is difficult. One question that arises for truncation is where such species are generated. α-Synuclein is predominantly degraded by lysosomal pathways [133,134], including chaperone-mediated autophagy [135], and the lysosomal cathepsins are important in proteolysis. Therefore, some truncated species are found in the lysosomes and it seems unlikely that they would cause damage to the cell. However, α-synuclein is also a substrate for cytoplasmic calpains [136-139], which are therefore more likely to generate cytoplasmic toxic truncated species. Some detail is therefore needed to prove which truncated species mediate toxicity, if any of them in fact do.

Oxidative stress, including the neurotransmitter dopamine, has been linked to increased α-synuclein aggregation [89,140]. Dopamine itself may contribute to the toxic effects of α-synuclein *in vitro *[89], although such a mechanism cannot explain why non-dopaminergic neurons die early in the disease process. α-Synuclein expression can enhance sensitivity to oxidative and nitrative stressors [141,142], although it can also be protective in some situations [143]. In most of these situations, the role of aggregation is unclear.

In summary, α-synuclein has properties, including the potential for aggregation and post-translational modifications, which may influence its toxic effects. Whether these are required for toxicity is unclear, and some results still need to be resolved, for example for the work on S129 phosphorylation. However, there is a larger question, which is: what effects synuclein has on neurons that are responsible for its toxic effects?

### Mediators of α-synuclein toxicity in biological systems

Some of the relevant data from cellular systems has been reviewed previously [144] and will be discussed here in the context of examples across multiple models.

Presumably, α-synuclein might interact with other biomolecules to mediate toxicity. Because α-synuclein can associate with lipids, membranes are one possible target. *In vitro*, α-synuclein can form pore-like structures [145,146], and annular rings of synuclein have been isolated from the brains of patients with multiple system atrophy, a synucleinopathy [147]. Cells expressing α-synuclein have increased cation permeability [148] and vesicles prepared from cultured cells or isolated from the adrenal medulla show leakage of catecholamines [149]. These events may be consistent with the formation of non-specific pores or similar structures at the plasma membrane or at a vesicle surface.

Because α-synuclein binds synaptic vesicles, it is possible that synaptic transmission would be directly or indirectly a target of synuclein toxicity. One example of this comes from work showing that A30P α-synuclein alters exocytosis of catecholamine containing vesicles in primary cells and in chromaffin cells [150]. The effect here is probably at a late stage of the exocytosis, before vesicle membrane fusion [150].

Further evidence for an effect of α-synuclein on vesicle function that may mediate toxicity comes from suppressor screens in yeast [63]. In the same organism, such defects can be localized to a block in endoplasmic reticulum (ER)-golgi vesicular trafficking [151]. Supporting this idea, there is evidence of ER stress [87] and golgi fragmentation [152] in mammalian cell systems.

Overexpression of Rab1, a GTPase that influences vesicle dynamics, was able to at least partially rescue the toxic effects of α-synuclein in yeast, worms and in mammalian cells [151]. Therefore, some of the toxic effects of α-synuclein that are conserved across species involve damage to vesicular transport, which might express itself as damage to presynaptic vesicle release in a neuron.

There are also suggestions that other membranous organelles are affected by α-synuclein, including mitochondria [87,88,153]. Recent data suggests that a portion of α-synuclein can localize to mitochondria, at least under some conditions [154-157]. Supporting this are observations that α-synuclein expression increases cellular organismal sensitivity to rotenone, a mitochondrial complex I inhibitor [78,158]. Furthermore, intact mitochondrial function is required for a-synuclein toxicity in a yeast model, although it should also be noted that removal of mitochondria is also quite damaging in the same context [57]. The mechanism by which α-synuclein interacts with and causes damage to mitochondria is not fully resolved and, given the central role of mitochondria in apoptotic pathways, perhaps such effects are secondary to the induction of apoptosis. Increased levels of α-synuclein are reported to trigger apoptosis in various cell types [159-161]. Several apoptotic markers are also seen in yeast models of synuclein toxicity [59]. α-Synuclein toxicity can be rescued by caspase inhibitors or knock down of caspase-12 [87]. Activation of caspase-3 has been reported in transgenic mice [162] caspase-9 has been reported in viral models in mice [102] and rats [106]. However, these studies show only a few caspase positive cells, and so whether apoptosis is the only way in which cells expressing α-synuclein die remains unclear.

α-Synuclein can bind to the membranes of lysosomes [135] and inhibit lysosomal function [163] and chaperone-mediated autophagy [135]. Recent results suggest that CMA is implicated in the regulation of the transcription factor MEF2D and that this can be disrupted by expression of α-synuclein, leading to neuronal death [164]. As another example of misregulated protein turnover, α-synuclein (and specifically α-synuclein oligomers) can also inhibit the proteasome [81,88,163,165-167], although it is not clear if the predicted altered turnover of proteasome substrates occurs *in vivo *[168].

The general principle is that multiple systems can be affected by α-synuclein expression and that if there is a common theme between them, it is likely to be that α-synuclein can binds lipids. Several lines of evidence suggest that lipid binding can promote the formation of oligomers [115,145,169]. Therefore, this interpretation links a primary protein abnormality to cellular targets of the protein. As discussed elsewhere [144], determining which events are truly primary and which are secondary remains a challenge. Although this distinction is an intellectual problem, it may also be relevant for deciding which aspects of cell death to target if we want to limit the disease process in PD.

## Potential therapeutic approaches related to α-synuclein toxicity

One of the key questions here is to decide whether to try and target the protein or the process that mediates cellular damage. Both are attractive for different reasons, although both are also difficult (see figure 1 for where these might be utilized and Appendix 2 for the critical next steps).

If there were a pathogenic aggregated form of α-synuclein, then one tactic would be to target that species. If we propose that insoluble fibrils are toxic, then a 'fibril-buster' would be the way forward [reviewed in [111]], but if soluble oligomers damage cells then we would want to prevent their formation or encourage their turnover. As discussed above, both fibrils and oligomers can be found in different models and either alone, or both, could be toxic. For oligomers, the situation is more complicated if different oligomeric forms have different toxic properties [118], suggesting that we may need to be careful about *which *oligomers we target.

Alternatively, we could be agnostic about which species are important and try and decrease all α-synuclein expression. There are reports that increasing autophagy can help clear aggregation-prone proteins, including α-synuclein [170]. Antisense approaches might also be helpful, and have been reported to work in the rat [171] and mouse [172] brain. This approach is predicated on the idea that α-synuclein really is dispensable for CNS function in humans, as it appears to be in the mouse [28,30], but perhaps even a modest decrease in protein levels would be enough to decrease PD progression.

We might also try to change the modifications of α-synuclein, especially if these are specific for pathogenic forms. For example, example of PLK2 as a kinase for Ser129 [127] may provide a way to test the idea that phosphorylation at this residue is key for pathogenesis, if sufficiently specific kinase inhibitors can be developed. Again, assuming specificity can be achieved, it might be interesting to block other modifications such as truncation or nitrosylation – the latter might be part of the general rubric of anti-inflammatory approaches. However, such approaches would only be helpful if the modification is truly specific for the pathogenic form and makes an active contribution to cellular toxicity, ie is not a bystander in the process.

Finally, we may target one or more of the cellular effects of α-synuclein that are associated with toxicity. This might have the advantage of leaving the protein alone, which may be useful if it turns out that α-synuclein has a specific function in the human brain. The difficulty, of course, is in understanding why the protein is toxic, although the work with Rab1 [151,173] suggests that this is a tractable problem, at least in principle.

## Conclusion

Cell death is a significant part of the pathology of PD. Although the process is a mysterious, the prime suspect for a toxic protein is α-synuclein. Assuming toxicity does indeed result from aberrant forms of the protein, including increased expression of the normal gene, there are two major aspects that might be targeted therapeutically. First, the protein is prone to aggregate and anti-aggregative compounds, or approaches to simply limit net expression levels, may be helpful. Second, there are a number of molecular events that largely revolve around membrane or organelle interactions that may contribute to toxicity, and these too may be targeted therapeutically. Future work should be directed at exploring these possibilities as well as at developing models that have a stronger cell death signal, to more accurately represent the substantive loss of neurons seen in PD.

## Abbreviations

DLB/DLBD: Dementia with Lewy bodies/Diffuse Lewy Body Disease; ER: endoplasmic reticulum; L-DOPA: 3,4-dihydroxy-L-phenylalanine; PD: Parkinson disease.

## Competing interests

The author declares that they have no competing interests.

## Appendix 1: key observations

The role of α-synuclein in PD and related disease is highlighted by the convergence of pathological and genetic data. Because part of the pathological phenotype of PD involves cell death of neurons, particularly but not exclusively dopamine neurons in the substantia nigra pars compacta, this suggests that α-synuclein may be a toxic protein. The following key observations have been made in various experimental systems to support this contention:

- In pure *in vitro *assays, α-synuclein shows a lack of conformational restraint that tends to promote inappropriate aggregation. This can be enhanced by mutation, increasing concentration or any of several protein modifications associated with pathological deposition of the protein *in vivo*. α-Synuclein can also bind lipids and membranes *in vitro*

- In a variety of species, expression of α-synuclein can promote toxic events. These include organisms such as yeast, worms and flies, where no α-synuclein homologue is present, suggesting that irrespective of its normal function, the protein can be toxic.

- Data in mammalian cell culture also supports a toxic effect of α-synuclein, particularly to dopaminergic cells. Results in intact *in vivo *systems are mixed, with toxicity limited to the spinal cord in some transgenic mouse models and modest toxic effects to dopaminergic neurons using viral mediated overexpression in rodents and non-human primates.

- The mechanism(s) involved are currently unclear, but binding to several cellular membranes may contribute to toxic events.

## Appendix 2: critical next steps

The following critical issues need to be addressed before our understanding of α-synuclein pathobiology can be applied to therapeutic development:

- We need to better understand normal function of α-synuclein, such that we can assess both what role it might play in toxicity in the mammalian CNS and so we can highlight potential detrimental effects of limiting expression or function of the protein.

- We need to clearly identify which cellular pathways contribute to the pathological effects of the protein. Some great work has been performed in yeast models that highlight interruption of vesicle transport, but it is important now to establish what the analogous process is in neurons and whether this is sufficient to explain α-synuclein toxicity in this system.

- We need to develop models where there is a lesion that better approximates the severity of cell loss seen in human PD. This will allow for a more rigorous test of pathways involved in toxicity as the disease progresses. An accelerated time course would be helpful, and may be necessary, but the pathology should be similar to human PD in that nigral neurons should be affected at some point in the model but not necessarily first or exclusively.
